# “I Want to Be Stepping in More” - Professional Online Forum Moderators' Experiences of Supporting Individuals in a Suicide Crisis

**DOI:** 10.3389/fpsyt.2022.863509

**Published:** 2022-06-13

**Authors:** Amanda Perry, Andrea Lamont-Mills, Jan du Preez, Carol du Plessis

**Affiliations:** ^1^School of Psychology and Wellbeing, University of Southern Queensland, Toowoomba, QLD, Australia; ^2^Laidlaw College, Social of Social Practice, Auckland, New Zealand; ^3^School of Psychology and Wellbeing, University of Southern Queensland, Ipswich, QLD, Australia; ^4^Centre for Health, Institute of Resilient Regions, University of Southern Queensland, Springfield, QLD, Australia

**Keywords:** online health community, health professional, online moderator, suicide, suicidal behavior (SB), online forum

## Abstract

**Introduction:**

Individuals experiencing suicidal crises increasingly turn to online mental health forums for support. Support can come from peers but also from online moderators, many of whom are trained health professionals. Much is known about users' forum experiences; however, the experiences of professional moderators who work to keep users safe has been overlooked. The beneficial nature of online forums cannot be fully realized until there is a clearer understanding of both parties' participation. This study explored the experiences of professional online forum moderators engaged in suicide prevention.

**Materials and Methods:**

A purposive sample of professionally qualified moderators was recruited from three online mental health organizations. In-depth semi-structured, video-recorded interviews were conducted with 15 moderators (3 male, 12 female), to explore their experiences and perceptions of working in online suicide prevention spaces. Data was analyzed using inductive thematic analysis.

**Results:**

Five themes were identified related to the experiences and challenges for moderators. These were the sense of the unknown, the scope of the role, limitations of the written word, volume of tasks, and balancing individual vs. community needs.

**Discussion:**

Findings indicate that the professionally qualified moderator role is complex and multifaceted, with organizations failing to recognize these aspects. Organizations restrict moderators from using their full therapeutic skill set, limiting them to only identifying and re-directing at-risk users to crisis services. The benefits of moderated online forums could be enhanced by allowing moderators to use more of their skills. To facilitate this, *in-situ* research is needed that examines how moderators use their skills to identify at-risk users.

## Introduction

Suicide is a leading cause of death and disability globally (1) where for every death by suicide it is estimated that more than 20 people attempt suicide (2). While many individuals who attempt or die by suicide reach out for support prior to their attempt (3) it is noted that some individuals do not reach out even when help is available (4). For those who do reach out, some are unable to access the support they need (5). It is for this reason that many individuals in crisis are turning to online forums for support and help (6). On some forums moderators are present to ensure the safety of forum users (7). For individuals experiencing suicidal behaviors, the support afforded by online forums may be lifesaving (8). While there has been research that examines the benefits of online forums from the perspective of forum users (9), most of the work has focused on users of bereavement forums, where it was assumed that the perspectives of users could be different to those held by the forum moderators (10). Therefore, for the beneficial nature online forums to be fully realized, research is needed that focuses on the perspectives of online forum moderators, as well as the roles they play in keeping online users safe.

Online forums can be associated with formal health support services (e.g., ‘Side by Side', which is the online community of Mind, a UK based Mental Health Charity) or be part of larger informal online spaces (e.g., r/SuicideWatch on the social media platform Reddit). Online mental health forums are specifically focused on mental health issues and are spaces where individuals can freely discuss their mental health issues (9). Such forums are available 24/7 meaning that support can be accessed when it is needed most (11). Both general health and mental health forums are typically constructed around peers providing support and advice to each other (12), however some forums are also overseen by moderators (13).

Moderators are usually categorized as either professionally qualified or peers (14). Peer moderators are often unpaid volunteers, selected because they have lived experience of mental illness and they are encouraged to draw upon and share this experience in order to support their forum peers (15). Conversely, professionally qualified moderators (henceforth professional moderators) hold either a tertiary level qualification in communications or a mental health related field and/or have completed in-house training, and are typically paid for their moderation work (16). Professional moderators often engage in administrative functions such as welcoming new users, editing content, providing technical support, as well as directly engaging and offering support to users in distress, which most commonly involves referring them to crisis services (17). While both professional and peer moderators are tasked with ensuring the safety of online forum communities (14), little is known about the moderation role from the perspective of the moderator. A recent study that examined internet forums for suicide bereavement found that some users held concerns regarding the ability of peer moderators (also referred to as designated users) to provide distressed users with appropriate and adequate support (10). The study further identified the need to ascertain if forum users and moderators held the same or different perceptions of what constitutes positive or negative forum experiences (10).

A scoping review undertaken by Perry et al. (18) that examined suicidal behaviors and moderator support in online mental health communities, found limited research focused specifically on moderator experiences. The research that they did identify focused on peer moderators (*n* = 4) rather than professional moderators (*n* = 1). The review noted that induction training requirements for peer moderators ranged from **2** days [see (19)] to **6** months [see (20)]. Furthermore, to fulfill the role of peer moderator, peers were not required to have a mental health background or training, however, they were required to have some form of people helping experience, such as having worked in education, rehabilitation, or the military [see (20)]. The scoping review featured **one** study where a professional moderator was present and was identified through their credentials of ‘M.D' (medical doctor) [see (21)]. The study by Hsiung (21) did not state any recruitment or induction training requirements for the professional moderator.

Findings from the review suggested that due to differing tasks and level of responsibilities between peer and professional moderators, it is not possible to generalize findings from peer moderators to professional moderators and vice-a-versa. For example, peer moderators were required to contact a clinical supervisor when at-risk forum users were identified [see (23)], whereas professional moderators did not have to escalate at-risk users to supervisors for instruction on how to support forums users in crisis [see (21)]. Therefore, because of their roles, professional and peer moderators have different levels of responsibility when it comes to interacting with forum users who are experiencing a suicidal crisis.

While there is some understanding of the beneficial nature of online mental health forums from the perspective of forum users (9), the experiences and perceptions of professional moderators who work within these forums to keep users safe, has been overlooked. Understanding the role of professional moderators within these forums will aid in understanding how moderators act within these forums in order to meet users' needs. It will also allow for the identification of challenges, missed opportunities and additional possibilities within this online space. Ultimately, understanding the work that is done by professional moderators will assist in ensuring that the online spaces that are created by suicide prevention organizations are safe spaces for individuals experiencing mental health difficulties. It will also assist these organizations in providing appropriate training to future professional online moderators.

In this study we sought to explore suicide prevention on online forums from the perspective of professionally qualified moderators through a series of qualitative, semi-structured interviews. Within this study suicide prevention is defined as the identification of risk and subsequent interventions employed to prevent an at-risk individual from moving from suicidal ideation to suicide attempt (22). We chose to specifically focus on professional moderators, because of the different skills that these moderators bring to the online space (18); the different ways they can work with individuals who are in crisis compared to peer moderators (23); and the different responsibilities they may have toward users in preventing suicide. As demand for online support increases, it is likely that more qualified health professionals will be working in online mental health forums in the future to meet this increased user demand. For this reason, it is critical that we understand not just what professional moderators do, but how they experience and perceive their role in online suicide prevention. Given that working with suicidal individuals face-to-face is seen as a demanding, challenging, and at times a confronting task (24), we expect working in the online environment will bring its own unique experiences and challenges. Therefore, the aim of our study was to explore the experiences and challenges that professional moderators encounter when interacting with users who are experiencing suicidal behaviors.

## Materials and Methods

### Study Setting

This study was a qualitative, interview, collective case study. A collective case study consists of more than one case with data coming from several different sources or individuals who are located at different sites (25). A collective case study allows for an increase in generalization of findings because data analytic findings are able to be compared both within and between cases (26). Thus, a collective case study was deemed appropriate to best capture the contextual aspects of professional online moderator work. Data was collected using semi-structured interviews that were completed between October 2019 and February 2020 using online Zoom software (27), and included participants from the United Kingdom, Canada, Australia, and New Zealand. Participants were employed at one of three online mental health organizations that conduct online forums that center upon communication via the written word, with forums being underpinned by the principles of peer support. This means that peers are the predominant form of support available to forum users, with moderators acting to identify risk and redirect those at risk to crisis services such as local Mental Health Crisis Teams. To be consistent with ethics approval, the forum organizations have been discussed collectively and not individually named to ensure the confidentiality of moderators and the organizations.

The role of the forums was similar with a shared focus on providing mental health support and engagement in suicide prevention practices through the identification of risk and subsequent intervention; however, none of the organizations were crisis services. At the time of this study, the organizations had been operating for between 8 and 22 years. The average number of yearly posts ranged from 69,365 to 240,000, and the average number of yearly page views ranged from 960,000 to 3.96 million views.

The official roles of the moderators who oversaw each of the three forums ranged from working in the background of the forum to review and monitor user posted content to ensure a safe online space; to assisting and supporting people to find and engage with appropriate support services; to interceding when conflict amongst users is present; and providing empathetic support through online listening, questioning and engagement. According to the participants of this study, they received in-house training provided by the organization that employed them. The training consisted of reading forum handbooks and completing shifts where they ‘shadowed' or observed the practices of an experienced moderator at work, until they eventually began undertaking the moderator tasks themselves.

### Participants and Recruitment

Participants were recruited via purposive sampling. The first author had worked as an online moderator for 5 years and was aware of online mental health organizations that employed professional moderators to oversee forums. Three organizations who provide online mental health forums that support individuals who experience suicidal behaviors were contacted via email to introduce the research project, and to ask permission to invite moderators to participate in this study. All three organizations agreed to provide the research team with access to their moderators, by way of forwarding a research invitation email to moderators in their employment.

Moderators who were interested in participating in the research were required to email the first author, who replied with a Participation Information Sheet and Consent Form that was completed and returned prior to the interview commencing. A total of 15 participants (3 male, 12 female) were recruited from the United Kingdom (*n* = 6), Australia (*n* = 5), New Zealand (*n* = 3), and Canada (*n* = 1). Two of the participants had previously been moderators and had assumed management responsibilities and thus were considered to still be able to speak to their experiences as moderators. The remaining 13 participants were working as moderators at the time of the interview. All participants held a tertiary level qualification in a relevant field (i.e., counseling, psychology, social work, or communications), and had completed an in-house programme of training that was specific to the forum they worked for. All were paid for their moderation work. Most of the participants (*n* =10) were registered with a professional health governing body (i.e., British Association for Counseling and Psychotherapy or the New Zealand Christian Counsellors Association). The average time working as a professional moderator was 3 years, with time in the position ranging from 3 months to 10 years. Participation was voluntary and at the completion of the data collection phase, participants who had completed the interview in their own time, as opposed to completing the interview as part of their work duties, were sent the equivalent of a NZ$20 gift card as a thank you for their participation.

### Procedure

After gaining informed consent, dates and times for the semi-structured interview were negotiated and finalized between the participant and the first author. The first author conducted and recorded all the interviews. A conversational style of interviewing was utilized to gather insights into participants' experiences and perceptions (28). An interview schedule was used as a guide to ensure that all aspects of the topic were discussed, while also providing flexibility to explore unanticipated content (29). Questions included: How do you know when a user is feeling suicidal (i.e., what gives it away?) How do you know what to do when engaging with a user who is feeling suicidal? From your experience, what are the challenges of engaging with users who are feeling suicidal? A copy of the interview schedule is in Supplementary File 1.

Due to the potential sensitivity surrounding suicide, a follow-up email was sent to each participant 1 week after their interview enquiring about their well-being and thanking them again for their participation. The recorded interviews were confidentially transcribed verbatim by a professional transcription company. As the three forum organizations represented in this study offer anonymity to forum users and moderators, participant pseudonyms were generated using the Masterpiece Name Generator website^1^

### Analysis Strategy

This study used Braun and Clarke (30) thematic analysis as the analytic approach. Initially, the first author read and re-read each transcript several times in order to become familiar with the data, making notes of repeating content or initial analytic ideas. At the next stage of analysis, codes were generated and assigned to the data. Codes were then grouped together to identify patterns or themes in the data, with the first author comparing within and across themes to identify the essence of each theme and to propose candidate names for the themes. Next, the first author reviewed the themes to ensure that all data allocated to each theme was consistent with the essence of the theme. As a part of this, quotes that best reflected each theme were identified to be included in the report. All authors then reviewed and discussed the themes over several research meetings before confirming and naming them. At this point, the writing up of the report began. To ensure the appropriate rigor and reliability, the ‘15-Point Checklist of Criteria for Good Thematic Analysis' (30) was used throughout the course of this study to guide the progression of the research.

### Ethics

The study was approved by the University Ethics Committee where the authors are located prior to data collection commencing.

## Results

Five themes were identified from the data that relate to core aspects of working to support users experiencing suicidal behaviors. They reflect the complex working environment of professional moderators. Themes were the unknown; constraints of the moderator role; limitations of the written word; the volume of tasks; and balancing individual and community needs. These themes are sequentially presented with illustrative quotations.

### Theme 1: The Unknown

Participants perceived their moderation work to occur within highly uncertain environments. Almost all participants referred to a sense of the unknown, uncertainty, and unsureness that permeated many aspects of their moderator work. The unknown often began with not knowing the identities of users due to the anonymous nature of the forums which whilst being seen as an essential feature of the service for users, bought with it, its own work challenges for the participants.

*It can be very difficult not knowing and that's what I've always struggled to sit with the not knowing. With the moderator role there is a greater sense of not knowing because of the anonymity of the site, and sometimes it can be hard to sit with that*. (Beatrice)

The unknown was also be felt as uncertainty regarding whether a user responded honestly to moderator questions about their present level of safety. Participants shared how in some situations they felt uncertain of how to respond to forum users in a way that would be useful for the user but would also provide the information that participants needed to ascertain risk, and therefore assess the safety of the user.

*One aspect when you sort of immediately see something and it feels a bit overwhelming, oh God, how - how do I respond to this? I'm not quite sure how to approach it*. (Clare)

The not knowing also extended to the actions undertaken by participants to support individuals in crisis. Participants often did not know if they had made a difference to the forum user experiencing a suicidal crisis, or whether the forum user had connected with suggested crisis services, which further contributed to the sense of the unknown that surrounds their online working environment. This not knowing was seen by participants as the result of moderator shift changes occurring during suicidal crises, emergency services not reporting outcomes back to the forum organization, forum users not sharing outcomes, or users not returning to the forum. In all instances the multiplicities of the unknowns and related uncertainties were framed by participants as challenging; something that could be overwhelming and have a negative impact.

*It's human nature to wonder and to sort of want more and sometimes people don't give that or they won't return or you'll think the worst. That can lead you to make assumptions, but, it's just not a space where you can do that, otherwise it will become too overwhelming, you have to take what you've got in this space*. (Freya)

The struggle associated with the sense of unknown was exacerbated for participants due to the nature of a suicidal crisis being a matter of life and death for the individual in the crisis. The participants expressed a deep sense of responsibility for keeping users safe whilst acknowledging the difficulties associated with not knowing if they have, in fact, kept them safe.

*That'd be the hardest bit because we just, we just don't have a picture and I'm sure that inevitably we have had people that have logged onto the forums and have died by suicide like statistically. We don't know. So that can be challenging*. (Anna)

### Theme 2: Constraints of the Role

Many of the participants perceived the moderation role as a constrained role, due to forum users wanting more support from moderators than what moderators are permitted by forum organizations to offer. The participants explained that professional moderators employed in peer support focused forums are not intended to be the main form of support for users, as peers are meant to provide support to one another. Instead, moderators work in the background to make risk assessments and re-direct at-risk users to formal crisis services; they do not provide direct crisis care themselves. This sense of constraint was experienced as a tension between participants wanting to provide the psychological support that they are qualified to offer people experiencing a suicidal crisis, and the limited parameters of their moderator role which are defined by the organization that employs them.

*We do fall into members feeling or seeming dependent on us, and often they're not able to access supports outside of the forum for whatever reason, whether it's financial or geographical, and so they come to us looking for that support and it can be hard not to want to provide it, but unfortunately the format doesn't allow that*. (Maria)

The constrained moderator role was further experienced by participants as challenging. It was seen to be difficult to put professional skills aside and not help users in crisis in the way the forum user wanted to be helped, knowing that they have the very skills to meet the user's needs. This was seen as especially problematic when considering that a large portion of professional moderator work consists of identifying crises and then re-directing at-risk users to external crisis services. Anna, who held a leadership position with a forum organization but had worked for 2 years as a moderator, shared “*It's really hard with moderation because we actually have to train them (moderators) to kind of scale back a lot of their skills.”*

One participant spoke specifically to this challenge, of wanting to do more for forum users who had previously felt unsupported by crisis services and was therefore reluctant to reach out again. Not being able to offer crisis support left the participant wanting to reach out and support the forum user in the way that the user wanted.

*I was just supporting someone who had spent hours today with the crisis team on the phone and was describing how they had a plan and intention, not immediate, we found out afterwards, but a plan and intention, and my best suggestion was to send them back to that same crisis team that they felt let them down. It just feels like I don't have much control, like what good am I able to offer them, right then? And then that's when I want to be stepping in more*. (Maria)

What became evident in the data is that many participants experienced a discrepancy between what they as professional moderators wanted to be able to offer forum user experiencing a suicidal crisis or behaviors and the constraining nature of their role.

### Theme 3: Limitations of the Written Word

The participants in this study communicated with forum users via the written word. Users textually posted on forums with moderators responding to these posts with textual replies. Most participants perceived that using only the written word was limiting as without verbal and visual cues, they felt their meaning-making was comprised, which is problematic given the potentially high stakes involved with assessing individuals who may be in a suicidal crisis. The limitations of communicating using only the written word were experienced by participants as a particular challenge when English is perceived to not be the first language of a user, and in times of user distress when coherency of the message can be compromised and potentially misinterpreted. This sense of compromise was seen as negatively impacting upon the participant's ability to do their job and therefore their ability to keep forum users safe. Put simply, if moderators cannot make sense of the posted messages of users, they can struggle to confidently determine whether risk to safety is present for the user and if so, the degree of risk. Participants also reported difficulties in their own efforts to communicate in clear and impactful ways with users to assess risk, and if needed, direct distressed users to official crisis supports. As participant Jasmine shared “*Some of the challenge is sitting with the nuance of what I can write that could make a difference in the way they see both life and death.”* Here the written word was perceived as limiting for moderators, with the limitation having the potential to significantly impact the safety of the user.

*How do you hold all of that and react in a way that is sort of calm, holding, and measured but gets them all the information they need to hopefully get themselves safe as soon as possible? It feels like a lot goes on in those moments and I always feel in those moments like everything we say really matters. Every sentence you type when you respond to a member in that stage really matters*. (Ivan)

Participants also spoke of balancing the need to communicate enough information to keep a user safe, while also not overloading the user, and potentially causing overwhelm and risk their disengagement with the moderator or the forum. This was conveyed as requiring skill and discernment on the part of the moderator, which at times of high user posting on the forum or multiple instances of at-risk user presentations, made for a pressurized and stressful moderation experience.

### Theme 4: The Volume of Tasks

All participants perceived there to be an overwhelming amount of forum work at times that was complicated by the need to complete a range of differing moderation tasks simultaneously.

*The forums are very, very busy, and there's lots of posts kind of coming through, lots of traffic on the forums, at the same time, there's breaches of guidelines or suicide risk, and just being able to kind of multitask and manage those things together*. (Helen)

These tasks range from the menial (e.g., checking if post content contains words from a predetermined list of words that may or may not indicate risk), to the enforcement of the forum house rules that govern safe practices, to responding to users in suicidal crisis. These tasks were perceived to draw upon different moderator skill sets such as technical skills to complete the tasks, procedural knowledge of the forum and organization, and also clinical skills to determine which actions may be required. Thus, it was a multiplicity of tasks, skill sets, and knowledges that were being activated at any one time and in any combination. The number of tasks engaged in was seen as further complicated by the need to be constantly alert and responsive to risk.

*We moderate every single post. When there's lots of posts that aren't really risky, aren't really breaching guidelines, and we're kind of spending time looking at that, rather than paying more attention and focus to the ones that do demonstrate risk or are breaching our guidelines. It can be quite time-consuming with the one moderator, and it can be hard and busy, and you're trying to balance all of that. It can be hard*. (Lottie)

The sheer volume of work was experienced by participants as a source of stress and pressure, not just in terms of the number of tasks, but also in relation to the variety of different tasks that must be simultaneously attended to. Some participants commented that the volume and diversity of tasks they are required to concurrently manage was one of the most challenging aspects of the professional moderator role. Several participants contrasted their moderator work to their face-to-face clinical work, highlighting the often-singular focus of their in-person therapeutic support in comparison to their moderator work which they saw as requiring a highly developed ability to multitask especially when working with risk.

*With one-to-one work you have a single focus in that moment. On the forum there's the multitasking aspect where it feels like you can be dealing with so many different varied issues at once and you're multitasking a bunch of other different things too. I really enjoy the work and I think we do a lot of good. But I do think it is an inherently stressful job. Probably more so than I gave it credit for when I started*. (Ivan)

### Theme 5: Balancing Needs

All participants referred to the tension of balancing user disclosure with forum safety. To seek help, forum users must disclose their suicidal crisis, however, there is an inherent tension associated with this disclosure that becomes relevant for the professional moderator. If users do this and disclose too much information to the wider forum community, their posted content may be, albeit reluctantly edited by the moderator. Moderators edit or remove content to keep the wider forum community safe, to prevent other users from potentially being triggered and therefore becoming unsafe. This relates to the concept of contagion where the sharing of content such as specific methods or means to end one's life may cause distress and heightened risk in another individual (31). However, participants also spoke of the right of users to share their own experiences without censorship. All participants demonstrated an understanding that as moderators they play a key role in keeping people safe in the forums, however, this comes with the need to balance the right of individual expression with the safety of the wider community of forum users.

*There's great freedom in online communication and just being able to put things out, but we definitely work with the community in reminding them, that when you're putting things out there, there needs to be some safety around how you do that*. (Shona)

Participants experienced the balancing of this individual right to expression and collective safety needs as a tension, beginning with the decision of, if and then when, or when not to moderate the content. The editing or removing a forum users' posted content was described by some participants as a personally difficult and challenging task.

*I think this is one of the biggest challenges for me because it feels so invasive. They very specifically have chosen words that express something inside whether it is intentional or whether it is a process of intent. Their intent is very seldom to hurt anyone else and so when we edit, we do that because we let them know that it could actually hurt someone else. And even though we know that that's not the intent, and even though they know we know that it, I think it is incredibly shaming, and I find that part of the process really, really, difficult*. (Jasmine)

This was attributed to participant perception that the posting of content on a forum often requires a lot of courage on the part of forum users to share their distress and is recognized as an essential step in reaching out for the support they may desperately need. This was further compacted by the feeling that editing or removal of content was often interpreted by the posting forum user to represent a moderator's intention to silence or reject them. Participants reported that often users are not expecting to be silenced or rejected when reaching out for support in an online mental health forum.

*There are instances where editing, I think, is a lot more challenging. And we kind of need to balance, allowing the member to tell their story and have that heard, which I think is extremely validating. And having it as much in their own words as possible. But then having to silence them somewhat by moderating or editing their post. Or sometimes removing parts of it, or all of it, in order for their post to fit within the house rules. So, although it's a simple, practical task I find it the most challenging*. (Heidi)

Participants shared that users whose content had been moderated could respond in anger directed at the moderator, as the forum user is often unable to see how their post and its content may cause distress to others. Alternatively, participants spoke of how forum users reacted with a shame response and retreated from the community after having their posts edited or removed. For some participants, forum users' responses of anger, shame, or retreating caused personal discomfort and were described as having a negative impact on them.

*You can imagine if you're feeling really vulnerable and exposed and then your first response from that service provider is, “Sorry, we can't have your narrative. It's too triggering for other members.” I literally had an email this morning where he used the phrase ‘disheartened, disappointed' and he just said he wouldn't come back to the service*. (Beatrice)

Participants appeared to deeply feel the therapeutic disconnect between moderating content and their professional understanding of how important it is for individuals to disclose how they are feeling, in order to get the support that they need. Participants felt that balancing forum community safety needs over the individual right to expression, and therefore moderating content, could have unintended consequences for individual forum users in terms of their willingness to reach out and seek help again in the future.

## Discussion

The study sought to explore the experiences and challenges that professional moderators encounter when interacting with users who are experiencing suicidal behaviors. What is clear from the results is that participants experience the moderator role as one that is multifaceted, complex, and constrained. An implication of the complexity and constraint included the impact that the professional moderator role had on participants, in terms of the challenge of sitting with the unknown when it came to the identity of users, the severity of risk presented, and the outcome of crises. For some participants it was identified as a potentially overwhelming aspect of their role if it was not carefully managed by the moderator. Additionally, this study showed that the participants wanted to be able to do more within the moderator role to support forum users in crisis. Professional moderators ensure forum safety but do so in constrained ways that often leave them wishing they could be stepping in more to support those in crisis. As qualified health practitioners the professional moderators have the clinical skills to support users in crisis but are unable to use these skills due to the remit of the mental health forums not being crisis forums. Instead of using their clinical skills as other clinicians would when working with individuals in crisis, they are limited to referring users to crisis services. Given the complexity of the multiple unknowns that moderators must manage and the wish to be able to use more of their skills, we propose that with more people turning to online spaces for support, particularly during the COVID-19 pandemic (32), the experiences of professional moderators must be a central consideration for improving online suicide prevention practices and research.

An unexpected finding from this study was the toll that forum moderation work can take on professional moderators. Such a toll and burden has been identified for peer moderators (33), but has not been previously considered for professional moderators. Given that the burdens identified in this study were associated with perceptions of distressed users not getting the support that they need (33), this finding has implications for the perceived benefits of online suicide prevention forums. In this study, this toll was evident in the tension that participants felt between encouraging those in distress to reach out for much-needed support using their own voice, and therefore their own words, while also working to ensure the overall safety of the forum community was not negatively impacted. This means that if an in-crisis forum user posted content that had the potential to cause other forum users to become unsafe, the content could be edited or removed by the moderators. The tension of having to edit an individual's content for the ‘greater good' of the forum community was felt on both professional and personal levels.

A possible explanation for this tension is that health professionals enter their profession to help those in need (34), and they are not often required to censor expressions of distress as can be required when working in online forums. Indeed, it is the role of the health professional during a consultation to encourage client exploration and give voice to that distress in ways in which the client feels best suits their needs (35). Given the public nature of online work and the impact editing content can have on moderators and forum users, future research should seek to examine the efficacy of current moderator editing practices with respect to reducing factors that increase suicide risk. Moderators noted that when they edited content to avoid other forum users potentially becoming unsafe, they felt this was invasive with some users finding moderator edits problematic. More research is needed to identify whether editing content that could be considered triggering for other users, exasperates risk or if it is preventative as intended.

Findings could help to identify alternative practices that maintain forum safety whilst also minimizing the negative impacts of such editing for both forum users and moderators. Such research may be in exploring whether the therapeutic alliance exists between a moderator and a user in crisis and if it does, how is this developed and maintained in online forums spaces and how is it ruptured and repaired. Client perceptions of the therapeutic alliance associated with e-mental health interventions has been found to be high and equivalent to face-to-face therapy (36). More importantly for the current study, this perception does not seem to be impacted by communication mode or the amount of contact between therapist and client. Alternatively, given the body of research identifying the ability to freely express thoughts, feelings, and plans online as a reason why individuals with lived experience of suicidal behaviors are attracted to online spaces (37), this tension felt by participants may be reflective of this. It is the lived disconnect between what users want and what some forums can offer.

A unique finding from this study was that while the participants may have been hired by the forum organizations for their professional skills, with their presence as qualified health practitioners promoted on forum websites as a means of creating a safer online forum experience for users, their response to those in crisis is limited by the organization to identifying and redirecting at-risk users to crisis services. While this may not necessarily be a problem for the forum organizations *per se*, the issue is that when there is a referral to an external service it is not always known whether the referred forum user takes up the referral and encouragement to seek external support. The participants shared that often users reported not wanting to seek help external to the forum, with referrals to crisis services potentially becoming barriers to getting help.

What this means for forum organizations is that they are failing to use the full skill sets of professional moderators. This is due to online forums not being a crisis service *per se*, despite an increasing number of individuals in crisis turning to online forums for support (38). A possible explanation is that while moderators may make the forums safer (i.e., by removing challenging content or checking in with users to assess their safety), it is potentially not in the way that the forum users may want or expect. This finding also signals a possible incongruence between what is promoted by the online forum organizations and the support that is available to forum users. This raises questions, not answered by this study, as to whether there are missed opportunities for suicide prevention work on forums that are professionally moderated (39). Missed opportunities in terms of clinically trained professionals using more of their skill sets to help users in ways that users come to the forums seeking and even possibly expecting. It may be that forum organizations need to be clearer in their signaling of the purpose and intention of their forum, given the wide variety of online forums that users may be simultaneously using, where the understanding of which forum offers what, may become blurred when the user is in crisis.

Future research to advance the field should include *in-situ* research that examines how moderators use their skills to identify at-risk users. Engaging with practice-based experiences is needed to further innovate online forum support. Research findings could identify the professionally qualified moderator practices that are most effective in encouraging at-risk individuals to engage with crisis services and help to identify what support may be possible if professional moderators were permitted to use more of their professional skills. This information could be used to inform the training of both professional moderators and peer moderators, to the benefit and safety of the forum users. Furthermore, as the demand for online support is increasing, it is likely that more qualified professionals will be working in online forums in the future to meet user demand. For this reason, it is crucial that we understand what currently occurs in these spaces, how moderators are working with users to alleviate distress, and what may be possible for forum moderators in the future when it comes to keeping online users at risk of suicide safe.

The impact of professionally moderated forums could be enhanced by reviewing and adapting the scope of the professional moderator role, to allow these moderators to use more of their professional skills. This assertion is supported by research that shows online counseling to be as effective as in-person counseling practices (40, 41), indicating that moderators asking to use more of their skills to be both reasonable and possible. The authors acknowledge that any changes to the scope of the professional moderator role may bring a range of ethical and practical challenges that are likely to require careful consideration. Practically, this may begin with considering how face-to-face skills translate to online text-based crisis work. For instance how will the lack of visual cues that are greatly valued in face-to-face crisis work be managed when working with crisis online (42). An ethical challenge may include considering how moderators employed by forums that serve users from a number of states or countries, will comply with the geographical restrictions of their practicing licenses (43). These licenses tend to limit practitioners to working with individuals who reside in the state or country of where they are licensed (44). An additional ethical concern includes how moderators would balance the anonymity that users value, with the requirements of moderators to avoid dual relationships. A dual relationship would not be possible to identify without knowing the identity of the user (43). Just as it would be difficult to overcome cultural blindness that can occur from not knowing the identity or other identifying information of a user (43).

This study has several strengths and weaknesses. It featured 15 participants from three online mental health forum organizations, with the results suggesting that there are similarities of professional experience across, as well as within, different organizations. This indicates that the experiences of professional moderators engaged in online suicide prevention reported here may be more generalizable than expected. Given the assertion that more qualitative studies in the field of suicidology are required to move the field forward (45), taking a qualitative approach to understanding professionally qualified moderator experiences is a strength of this study in that it provides an in-depth insight into the challenges of engaging in online suicide prevention. Moreover, it does that from the moderator perspective rather than the forum user, bringing new insights and potential learnings to online suicide prevention practice and research.

A further strength of this study was the utilization of thematic analysis. As a methodology, thematic analysis offers an highly flexible approach to analyzing study data (46) that can be useful in examining the perspectives of different research participants, to highlight the similarities and differences that exist, and to generate unanticipated insights. The disadvantages of using thematic analysis include the inability of researchers to infer meaning associated with the language use of research participants, as well as the lack of supporting thematic analysis literature when compared to other qualitative research methods, which can inhibit the ability and confidence of inexperienced researchers in conducting rigorous analysis (47).

A further limitation of this study was that it featured professional moderators from four English speaking countries, which means the themes identified in this study may not be relevant or applicable to professional moderators in non-English speaking countries. This is due to forums of English speaking countries being more likely to reflect Western perspectives of suicide, that may not align with non-Western perspectives. For example, in English speaking countries suicide is more often attributed to mental disorders than in non-English speaking countries (48). In non-English speaking countries, psychosocial stress and social isolation rather than mental disorders are deemed the predominant factors for suicide (49). For this reason, professional moderators in non-English speaking countries may experience different challenges when it comes to supporting forum users in crisis.

## Conclusion

This study illustrates the experiences of professional forum moderators when working to support individuals experiencing a suicidal crisis online. We suggest that professional moderators have skill sets that are not fully utilized by the forum organizations that employ them, indicating that professional moderators are both willing to, and are capable of, doing more when it comes to online suicide prevention. Given that people in crisis are increasingly turning to online forums for support, regardless of whether forum organizations provide crisis services or not, our findings suggest that it may be time for forum organizations to reconsider their support models. Our findings help to establish a body of literature and encourage practitioners, researchers, and policymakers to engage with practice-based experiences to further innovate online support for those in crisis. Reviewing and adapting the scope of the professional moderator role may be a key element to harnessing the full potential of online forum support, with *in-situ* research needed that examines moderator practices to identify what currently happens and what may be possible in the future.

## Data Availability Statement

The datasets presented in this article are not readily available because of the potentially identifiable nature of the data. Requests to access the datasets should be directed to amanda.perry@usq.edu.au

## Ethics Statement

The studies involving human participants were reviewed and approved by the University of Southern Queensland Ethics Committee. The patients/participants provided their written informed consent to participate in this study. Written informed consent was obtained from the individual(s) for the publication of any potentially identifiable images or data included in this article.

## Author Contributions

AP, AL-M, JdP, and CdP contributed to the conception and design of the study. AP conducted the interviews and wrote the first draft of the manuscript. AL-M, JdP, and CdP provided guidance, supervision during the data analysis, and edited and contributed to the manuscript. All authors contributed to the article and approved the submitted version.

## Funding

The primary author is the recipient of a Research Training Program Stipend Scholarship.

## Conflict of Interest

The authors declare that the research was conducted in the absence of any commercial or financial relationships that could be construed as a potential conflict of interest.

## Publisher's Note

All claims expressed in this article are solely those of the authors and do not necessarily represent those of their affiliated organizations, or those of the publisher, the editors and the reviewers. Any product that may be evaluated in this article, or claim that may be made by its manufacturer, is not guaranteed or endorsed by the publisher.

## Footnotes

^1^ https://masterpiece-generator.org.uk
