# Supplementary material for: “I Want to Be Stepping in More” - Professional Online Forum Moderators' Experiences of Supporting Individuals in a Suicide Crisis
Source: Front Psychiatry. 2022 Jun 13;13:863509. doi: 10.3389/fpsyt.2022.863509 (PMC9238438; doi:10.3389/fpsyt.2022.863509)
Supplement: Supplementary file 1 [file Data_Sheet_1.PDF]

Interview Protocol Form

Title of Project: Suicidal behaviours and moderator support in online health communities

Human Research Ethics Approval Number: H19REA120 (v1)

Date ___________________________

Time ___________________________

Location ________________________

Interviewer ______________________

Interviewee ______________________

Interview Qualifications

Professional Body Affiliation:

Consent form signed? ____

Notes to interviewee:

Thank you for your participation. I believe your input will be valuable to this research.

Confidentiality of responses is guaranteed

Approximate length of interview: 60 minutes, 10 questions

Purpose of research:
The identify the perceived challenges of moderators, associated with the engagement and support of forum community members who are experiencing suicidal ideation.

Methods of disseminating results: Published article or thesis

1. **Take me back through the history in your career that brought you to be a moderator.**
   What are your qualifications?
   Which professional bodies are you associated with?
   How long (in months and years) have you worked as a moderator?

Response from Interviewee

Reflection by Interviewer

2. Can you tell me about are the most common mental health issues you encounter as a moderator?

You have said X, Y and Z. Are there any others?

Response from Interviewee:

Reflection by Interviewer

3. Tell me, what aspects of the moderator role do you find the most challenging and why?

Response from Interviewee

Reflection by Interviewer

4. You have mentioned that suicidal ideation is a common member presentation. How do you know when a member is feeling suicidal i.e. what gives it away?

Look for examples of behaviour from the moderator
Are the cues only in written form?
Are there any prompts from the system?

Response from Interviewee

Reflection by Interviewer

5. So once you have identified that a member is feeling suicidal, what happens next?

Consider the participants thoughts, assessments, and actions.

Response from Interviewee

Reflection by Interviewer

6. So I can hear that you can take a number of steps in support members who presented as feeling suicidal. How do you know what to do when engaging with a member who is feeling suicidal?

Look for previous experience
Training
Research based practice

Response from Interviewee

Reflection by Interviewer

7. From your experience, what are the challenges of engaging with members who are feeling suicidal?

Thinking about lack of visual cues
Anonymity
For the moderator personally

Response from Interviewee

Reflection by Interviewer

8. What support is available to a moderator when engaging with members who are feeling suicidal?

Consider support from the forum
Consider support from beyond the forum

Response from Interviewee

Reflection by Interviewer

9. Tell me, what aspects of the moderator role do you enjoy the most and why?

Response from Interviewee

Reflection by Interviewer

10. Is there anything else about your experience of the moderator that you would like to tell me?

Response from Interviewee

Reflection by Interviewer

- Closure
  - Thank you to interviewee
  - reassure confidentiality
  - ask permission to follow-up ______
